# Sodium butyrate interrupts the maturation of oocytes and enhances the development of preimplantation embryos

**DOI:** 10.1371/journal.pone.0220479

**Published:** 2019-07-29

**Authors:** Meng-Fei Yu, Ju-Long Wang, Jian-Ming Yi, Lin Ma

**Affiliations:** 1 Hubei Provincial Key Laboratory for Protection and Application of Special Plant Germplasm in Wuling Area of China, College of Life Sciences, South-Central University for Nationalities, Wuhan, Hubei, China; 2 Key Laboratory of State Ethnic Affairs Commission for Biological Technology, College of Life Sciences, South-Central University for Nationalities, Wuhan, Hubei, China; 3 Key Laboratory of Agricultural Animal Genetic, Breeding, and Reproduction for Ministry of Education, College of Animal Science, Huazhong Agricultural University, Wuhan, China; 4 School of Biotechnology, Wuhu Institute of Technology, Wuhu, China; 5 Key Laboratory of Aquatic Botany and Watershed Ecology, Wuhan Botanical Garden, Chinese Academy of Sciences, Moshan, Wuchang, Wuhan, Hubei, China; Friedrich-Loeffler-Institute, GERMANY

## Abstract

Histone acetylation is one of the most important posttranslational modifications that contribute to transcriptional initiation and chromatin remodeling. In the present study, we aimed to investigate the effect of sodium butyrate (NaBu), a natural histone deacetylase inhibitor (HDACi), on the maturation of oocytes, preimplantation embryonic development, and expression of important developmental genes. The results indicated that NaBu decreased the rates of GVBD and the first polar body extrusion (PBE) *in vitro* in a dose-dependent manner. Meanwhile, NaBu treatment led to an abnormality in the spindle apparatus in oocytes in MI. However, the ratio of phosphor-extracellular signal-regulated kinases (p-ERK)/ERK significantly decreased in oocytes treated with 2.0 mM NaBu for 8 h. Furthermore, NaBu treatment at 2.0 mM improved the quality of embryos and the mRNA expression levels of important developmental genes such as *HDAC1*, *Sox2*, and *Pou5f1*. These data suggest that although a high concentration NaBu will impede the meiosis of oocytes, 2.0 mM NaBu will promote the development of embryos *in vitro*. Further investigation is needed to clarify the direct/indirect effects of NaBu on the regulation of important developmental genes and their subsequent impacts on full-term development in mammals.

## Introduction

In mammals, histone modifications, such as acetylation, phosphorylation and methylation, sumoylation, ADP-ribosylation, and ubiquitination, play crucial roles in the development of oocytes/embryos [1–4]. Multiple histone modifications work in concert to regulate diverse biological functions, such as the cell cycle [5], DNA transcription [6, 7], and embryonic development [8]. Disruption of histone modification patterns can impair chromosome condensation and segregation, delay the progression of oocyte maturation, and even accelerate the aging of oocytes [9]. The acetylation patterns of oocytes vary widely depending on animal species. In general, histone acetylation is a lysine-specific process that occurs during the maturation of oocytes and functions in a meiosis stage-dependent manner.

Previous studies have reported that treatments with histone deacetylases inhibitors (HDACis) such as trichostatin A (TSA) [10], scriptaid [11], and valproic acid [12] lead to an increase in histone acetylation levels. An increased histone acetylation level in donor cells and/or cloned embryos is beneficial because it improves the developmental competence of embryos. For example, trichostatin A (TSA) treatment of embryos resulted in an increased rate of blastocyst formation in rabbits [13], pigs [14, 15], and cattle [16] and even live cloned offspring in mice [17]. Even in interspecies cloned embryos, TSA treatment can also improve the *in vitro* developmental competence of somatic cell nuclear transfer embryos [18–20]. However, some researchers reported that HDACi treatment exerted detrimental effects on the development of embryos [21, 22]. Taken together, the effects of an HDACi on embryonic development may vary depending on the type of HDACi and animal species.

Sodium butyrate (NaBu), a non-toxic 4-carbon fatty acid, can be synthesized by microbial metabolism in the colon [23]. NaBu can act as a non-competitive inhibitor of HDAC and does not associate with the substrate-binding site [24]. Although the binding site and mechanisms of NaBu-induced inhibition of HDAC activity remain unclear, many attempts have been made to explore the effects of NaBu on improving the development competence of oocytes and/or embryos in many species. For example, treatment of donor cells with NaBu significantly enhanced the rates of blastocyst formation in cattle [25] and rabbits [26]. NaBu treatment enhanced the developmental competence of interspecies bovine-yak SCNT embryos and “corrected” the expression patterns of important developmental genes [27]. However, Das et al. reported that NaBu treatment of cloned embryos rather than donor cells increased the developmental ability of preimplantation embryos in pigs [28]. The underlying mechanism for this discrepancy remains elusive.

The relation between NaBu and preimplantation embryonic developmental competence has been well documented. However, less attention has been paid to the effects of HDACi on the maturation of oocytes, especially germinal vesicle (GV) oocytes. This study mainly aimed to determine how NaBu affects the maturation of GV oocytes and the underlying mechanisms. In this study, the effects of NaBu on the rates of GVBD and the first polar body extrusion (PBE), spindle apparatus, and pluripotent gene expression were explored. Meanwhile, immunofluorescence was performed to determine the acetylation level of H3K9 (lysine residue 9 of histone H3), a hallmark of transcriptionally active chromatin, in oocytes and embryos derived from *in vivo*, *in vitro* fertilization (IVF) and parthenogenesis (Ps), respectively. Finally, the effects of NaBu on preimplantation embryo development were investigated.

## Materials and methods

### Reagents

All chemicals were purchased from Sigma-Aldrich Chemical Company (St. Louis, MO, U.S.A.), unless otherwise stated. Sterile cell culture plastic wares were purchased from Corning (Corning, NY).

### Animals

SPF Kunming mice were purchased from the Hubei Provincial Center for Disease Control and Prevention (Wuhan, China). All animals were housed in a temperature- and humidity-controlled room with artificial illumination (8:00 AM to 8:00 PM, lights on). The animals were given *ad libitum* access to food and water. All methods and experimental protocols involving animals were performed in strict accordance with the relevant guidelines and regulations of the Animal Care and Use Committee of Huazhong Agricultural University. This study was approved by this committee.

### Oocyte collection and culture

GV oocytes were isolated as previously described [29]. Briefly, female Kunming mice at 6–8 weeks of age were intraperitoneally injected with 10 IU pregnant mare serum gonadotropin (PMSG) and killed by cervical dislocation 48 h later. Cumulus-oocyte-complexes (COCs) covered with compact cumulus cells were collected by puncturing the antral follicles. After three washes in M2, the COCs were cultured in 100 μL M2 droplets supplemented with 0.4% BSA and 0.2 mM isobutylmethylxanthine (IBMX) at 37°C in 5% CO_2_ in air.

The superovulation and isolation of ovulated MII oocytes were performed as previously described [30]. Briefly, superovulation was induced in 6–8-week-old mice by an intraperitoneal injection of 10 IU PMSG followed by an intraperitoneal injection of 10 IU human chorionic gonadotropin (HCG) 48 h later. After 13 h, these mice were sacrificed to collect the ovulated oocytes from the oviductal ampullae. Then, the isolated COCs were cultured in potassium simplex optimized medium (KSOM) at 37°C in 5% CO_2_ in air.

### Mating and collection of *in vivo* fertilized eggs

Female mice were intraperitoneally injected with 10 IU PMSG and then injected with 10 IU HCG 48 h later as described above. After they were injected with HCG, female mice were individually mated with male mice. At 8:00 the next morning, females were checked for the presence of a vaginal plug, which indicated successful mating. Embryos at different stages were retrieved by flushing the oviducts using M2 medium supplemented with 3 mg/mL BSA before embryonic implantation according to the experimental design.

### *In vitro* fertilization (IVF)

Sperms were collected and capacitated as previously described [31]. Briefly, male mice were sacrificed with an intraperitoneal injection of 150 mg/Kg sodium barbital. Then, the cauda epididymides were isolated, and the contents were squeezed out for capacitation in human tubal fluid at 37°C for 1.5 h in an atmosphere of 5% CO_2_ in air.

COCs were collected as described above and transferred into 100 μL HTF droplets. Then, the capacitated sperms were added into these droplets to yield a motile sperm concentration of 1.5–2×10^6^/mL. After 6 h of co-incubation, these oocytes were transferred to M2 medium for culture.

### Parthenogenesis (Ps)

Mouse ovulated MII oocytes were isolated as described above [32]. The Ps of oocytes was performed as previously described. Briefly, freshly ovulated oocytes were obtained and cultured in KSOM supplemented with 3% BSA. After removal of the outer cumulus cells, they were equilibrated for 1 h in KSOM. Then, the oocytes were activated in 7% ethanol plus 5 μg/mL cytochalasin B for 5 min. Then, the activated oocytes were transferred into KSOM containing 3% BSA for further culture.

### Embryo culture

At 6 h after insemination, oocytes were transferred into M2 droplets containing 3 mg/mL BSA covered with mineral oil at 37°C in an atmosphere of 5% CO_2_ in air. Successful fertilization was confirmed by the presence of two pronuclei at 9–10 h after insemination. Then, the numbers of 2-cell, 4-cell, morula, and blastocyst embryos were counted at 18 h, 48 h, 72 h, and 96 h after insemination, respectively, according to the experimental design.

### Cell counting of blastocysts

Cell counting of blastocysts was performed as described previously with some modifications [33]. Briefly, the embryos derived from the IVF and NaBu+IVF groups were collected and stained with Hoechst 33342 for 10 min. Then, the images were obtained using a Fluoview 1000 laser scanning confocal microscope (Olympus Corp.) using a 20× objective. Z-stacks were taken for each embryo. Meanwhile, bright field images for each embryo were also taken to facilitate downstream cell counting. Cell counting was performed using Fluoview V10-ASW 2.1 software.

### Western blotting

Western blotting was performed as previously described with some modifications [34]. Briefly, dozens of mouse embryos were collected and heated in 2×SDS for 5 min at 100°C to obtain the total proteins. The proteins were separated by SDS-PAGE and transferred to PVDF membranes. After blocking in TBST containing 3% BSA for 2 h, the membranes were incubated with anti-phosphorylated ERK or anti-ERK antibodies (Cell Signaling Technology, Beverly, MA, USA) diluted 1:500 in blocking solution at 4°C overnight. After three washes in TBST, the membranes were incubated with HRP-conjugated goat anti-rabbit IgG for 2 h at 37°C. Finally, antibody binding was visualized and analyzed using Image-Pro Plus 6.0 software (Media Cybernetics, Silver Spring, MD, USA).

### Immunofluorescence

The immunofluorescence analysis and fluorescence intensity analysis were performed as previously described with some modifications [35, 36]. Briefly, embryos were fixed with 4% paraformaldehyde for 30 min, followed by permeabilization in 0.5% Triton X-100 for 30 min. After three washes in PBS containing 0.1% Tween 20, the embryos were blocked with 2% BSA in PBS for 1 h and incubated with the primary antibodies anti-AcH3K9 (1:200, Cat No: AB10812; Abcam, Cambridge, MA, U.S.A.) overnight at 4°C. Following three washes in PBS, the embryos were incubated with fluorescent isothiocyanate (FITC)-conjugated anti-rabbit secondary antibodies (1:100, Boster, Cat No: BA1105, Wuhan, China) for 1 h at 37°C. Finally, the DNA was counterstained with DAPI for 10 min. After washing three times, the embryos were observed using a Nikon Eclipse TE2000-U fluorescence inverted microscope equipped with a Nikon DS-Fi1 digital camera (Nikon, Tokyo, Japan). Fluorescence intensity was measured by analyzing the images with Image-Pro Plus 6.0 software (Media Cybernetics, Silver Spring, MD, USA). At least 10 embryos from each group were analyzed.

### Real-time PCR

The extraction of the total RNA of mouse embryos was performed in accordance with the manufacturer’s instructions (QIAGEN RNeasy Plus Micro Kit, Cat#74034). The concentration of RNA was measured using a NanoDrop 1000 spectrophotometer (Thermo Fisher Scientific, Wilmington, DE, USA). Then, 2 μg of RNA was reverse-transcripted into cDNA using a Side-Step QRT-PCR cDNA Synthesis Kit (Stratagene, LaJolla, CA) [37]. The detailed information on the primers is listed in **Table 1**. Primers were synthesized by Tsingke Biological Technology Co., Ltd., (Wuhan, China).

**Table 1:** Detailed information on the primers used in PCR.

Genes	Primer sequences (5ʹ-3ʹ)	Length (bp)	GenBank accession No.
*HDAC1*	F- CTGAATACAGCAAGCAGATGCAGAG	92	NM_008228
	R- TCCCGTGGACAACTGACAGAAC		
*Pou5f1*	F-GCCCTCCCTACAGCAGATCA	73	NM_013633.3
	R-GAACCATACTCGAACCACATCCTT		
*Sox2*	F-AAGACGCTCATGAAGAAGGATAAGTAC	76	NM_010411
	R-CGCTCGCCATGCTGTTC		
*β-actin*	F- CATCCGTAAAGACCTCTATGCCAAC	171	NM_007393.5
	R- ATGGAGCCACCGATCCACA		

### Experimental design

**Experiment 1:** Effects of NaBu on the *in vitro* maturation of GV oocytes.

Freshly isolated GV stage oocytes were randomly divided into six groups that were supplemented with 0, 0.1, 0.5, 1.0, 5.0, or 10.0 mM NaBu. After co-incubation in KSOM with NaBu at one of the six concentrations for 3 h, the rates of germinal vesical breakdown (GVBD) were calculated. Then, the oocytes were transferred into NaBu-free KSOM and cultured for 11 h. After the culture, the rate of oocytes that reached the MII stage (matured) was calculated. The completion of GVBD is marked by the disappearance of nuclear membrane oocytes, while the first PBE marks the maturation of the oocytes.

**Experiment 2:** Effects of NaBu on the formation of spindles in oocytes *in vitro*.

GV stage oocytes were isolated and randomly divided into three groups. In group 1, the GV stage oocytes were culture in NaBu-free KSOM for 8 h to the MI stage as a control. In group 2, the GV stage oocytes were first cultured in KSOM without NaBu for 3 h and then cultured in KSOM supplemented with 2.0 mM NaBu for 5 h. In group 3, the GV stage oocytes were continuously cultured in KSOM supplemented with 2.0 mM NaBu for 8 h. After the 8 h culture, the spindles of these presumptive MI oocytes were analyzed using an immunofluorescent method.

**Experiment 3:** Effects of NaBu on the expression of p-ERK/ERK in mouse oocyte meiosis.

Mouse GV oocytes were collected, divided, and treated as described above in experiment 2. The total proteins of oocytes in the three groups were extracted. Western blotting was used to analyze the effects of NaBu on p-ERK/ERK, which plays an important role in the maturation of oocytes.

**Experiment 4:** Effects of NaBu on dynamic changes in H3K9ac in mouse oocytes.

Mouse GV oocytes were collected and cultured to the GVBD, MI, and MII stages *in vitro* as described in experiment 1 in KSOM without/with 2.0 mM NaBu. After 3, 8, and 14 h of culture, GV oocytes will reach the GVBD, MI, and MII stages, respectively. Then, immunofluorescence was performed to measure the expression levels of H3K9ac in oocytes at these four different stages.

**Experiment 5:** Dynamic changes in H3K9ac in mouse embryos derived from *in vivo*, Ps, IVF, and IVF+NaBu.

Mouse GV oocytes were isolated and cultured in KSOM without/with 2 mM NaBu to the MII stage. The matured oocytes were used for IVF and cultured to the blastocyst stage. The embryos derived from *in vivo* and Ps were used as controls. These four types of embryos were collected and divided into five groups: the 2-cell, 4-cell, 8-cell, morula, and blastocyst stage groups. Immunofluorescence was performed to measure the expression of H3K9ac protein in these three types of mouse embryos. Therefore, the dynamic changes in H3K9 acetylation levels in embryos from cleavage to blastocyst were elucidated.

**Experiment design 6**: Effects of NaBu on early embryonic development *in vitro*. Mouse matured oocytes were collected and used for IVF. After fertilization, the oocytes were transferred into KSOM without/with 2.0 mM and cultured for 24 h. Then, these oocytes were transferred into KSOM and cultured to the blastocyst stage. The rates of embryos at the 2-cell, 4-cell, and blastocyst stages were calculated.

**Experiment 7:** Effects of NaBu on the mRNA expression of *HDAC1*, *Sox2*, and *Pou5f1*.

Mouse embryos derived from IVF at the 2-cell, 4-cell, and blastocyst stages as described above in experiment 6 were obtained and divided into two groups: the NaBu-free and IVF+NaBu groups. Embryos derived from natural mating were used as controls. The total RNA of these embryos was extracted and reverse transcribed. Then, real-time PCR was employed to measure the changes in the mRNA expression of the *Pou5f1*, *Sox2*, and *HDAC1* genes. Beta-actin was used as a loading control.

### Statistical analysis

At least three replicates were performed for each treatment. All percentage data were subjected to arcsine transformation before statistical analysis. All data were analyzed with SPSS (Statistics Production for Service Solution, Version 17.0) using one-way ANOVA, and differences between treatment groups were determined by Duncan’s multiple comparison test. Data are presented as the mean ± s.e.m., and differences at *P* < 0.05 were considered significant.

## Results

### Effects of NaBu on the maturation of mouse oocytes *in vitro*

**Fig 1:**
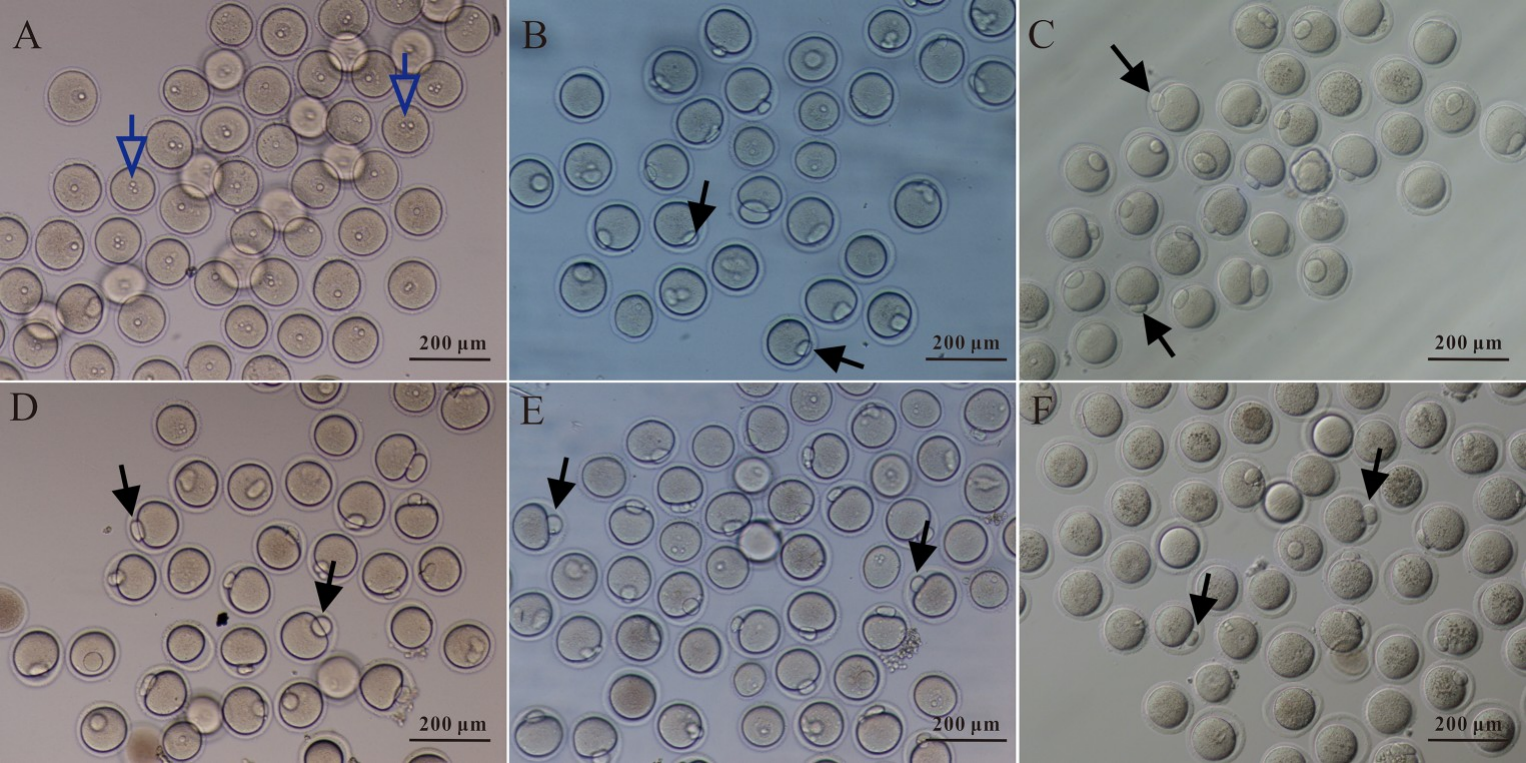
Effects of NaBu on the maturation of GV stage oocytes in vitro. Mouse GV stage oocytes were isolated (A) and cultured in KSOM supplemented with NaBu at different concentrations in vitro. Representative pictures of oocytes cultured for 14 h in KSOM supplemented with 0.1 mM (B), 0.5 mM (C), 1.0 mM (D), 5.0 mM (E), and 10.0 mM NaBu (F). Hollow arrows indicate the germinal vesical. Black arrows indicate the first polar body. Scale bars: 200 μM.

To investigate the effects of NaBu on the *in vitro* maturation of oocytes, the rates of GVBD and maturation of mouse GV stage oocytes were assessed. Mouse GV oocytes were isolated and cultured with NaBu at different concentrations (**Fig 1**). As shown in **Table 2**, NaBu at low doses (0.1 and 0.5 mM) did not alter the rates of either GVBD or maturation. Meanwhile, NaBu at high doses (1.0, 5.0, and 10.0 mM) inhibited the rates of both GVBD and maturation (*P* < 0.05). These data suggest that NaBu at high doses reduces the rates of GVBD and maturation of GV oocytes. Based on these results, NaBu at 2.0 mM was used in the following experiments.

**Table 2:** Effects of NaBu at different concentrations on the maturation rates of mouse GV stage oocytes.

Concentration (mM)	No. of oocytes	GVBD rate (%)	PBE rate (%)
0 mM	248	85.2 ± 3.8^a^	78.2 ± 5.7^a^
0.1 mM	213	84.1 ± 2.4^a^	76.1 ± 5.2^a^
0.5 mM	185	84.8 ± 2.0^a^	68.4 ± 3.7^a^
1.0 mM	243	77.1 ± 2.7^b^	55.2 ± 7.1^b^
5.0 mM	282	71.6 ± 2.2^b^	53.3 ± 2.9^b^
10.0 mM	189	70.7 ± 0.03^b^	30.2 ± 0.1^c^

Note: Values with different superscripts in the same column indicate significant differences (*P < 0*.*05*).

### Effects of NaBu on the spindle apparatus of *in vitro* matured oocytes

**Fig 2:**
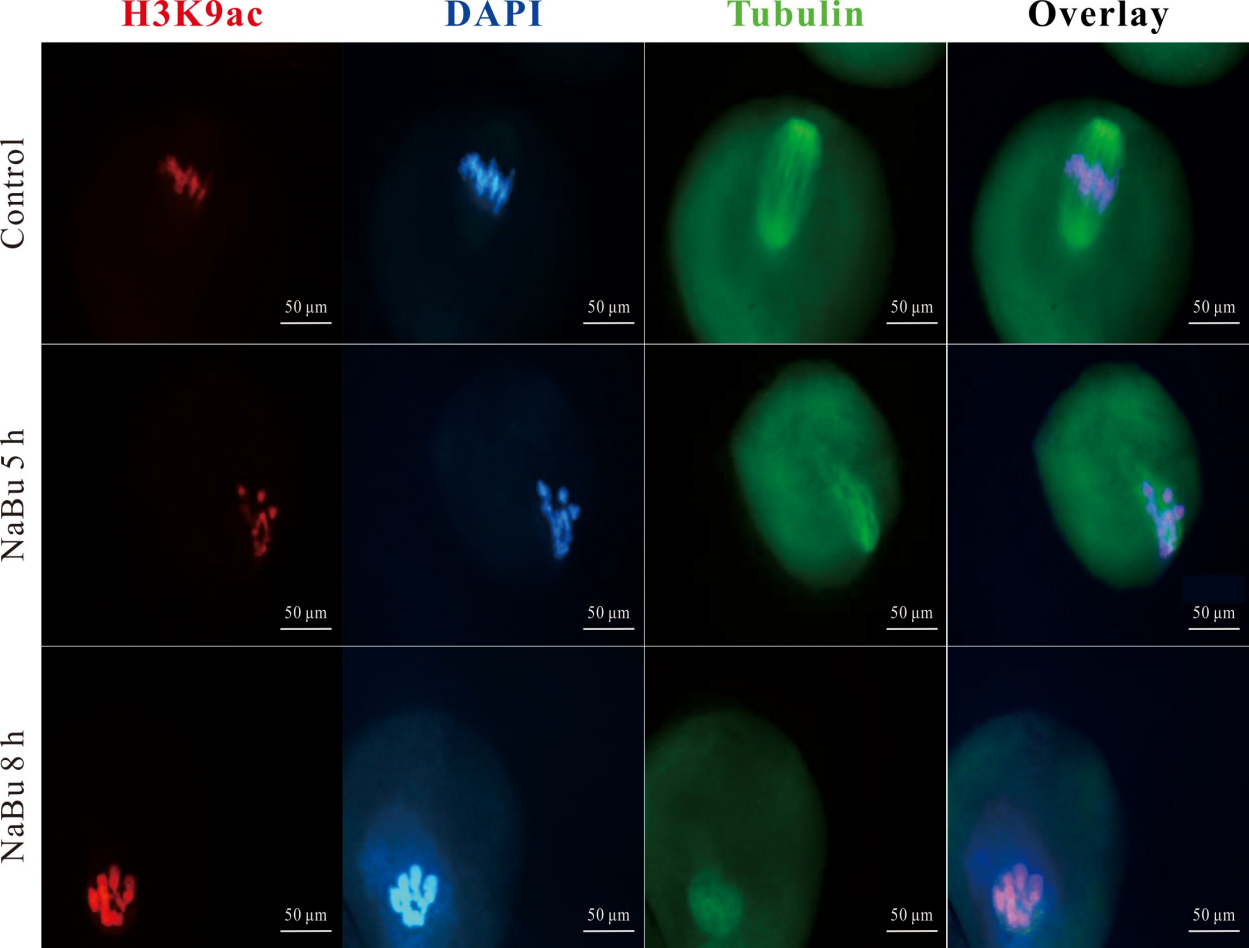
Effects of NaBu treatment on the spindle apparatus of MI stage oocytes. Mouse GV stage oocytes were obtained and randomized into three groups as described in experiment 2. After 8 h of culture, the oocytes were immunostained with anti-acetyl-histone H3K9 (red) and anti-α-tubulin (green) antibodies. The DNA of these oocytes was counterstained with DAPI (blue). Scale bar: 50 μM.

To further explore the effects of NaBu on oocyte maturation, the formation of the spindle apparatus of *in vitro* maturated oocytes was studied using immunofluorescence. As shown in **Fig 2**, in control group a, GV stage oocytes reached the MI stage after 8 h of *in vitro* culture. The fluorescent results indicated that oocyte chromosomes were aligned orderly and segregated towards the two spindle poles. In experimental group b, GV stage oocytes also reached the MI stage. However, the chromosomes of oocytes were distributed in an aberrant order, which interrupted the segregation toward the spindles. In experimental group c, the chromosomes of oocytes condensed, and the spindle twisted around the chromosomes and formed an oval shape. This irregular distribution of chromosomes and spindles markedly impeded the segregation of chromosomes. These results demonstrated that NaBu treatment can interrupt the formation of the spindle apparatus in *in vitro* matured oocytes.

### Effects of NaBu on the expression of p-ERK/ERK

**Fig 3:**
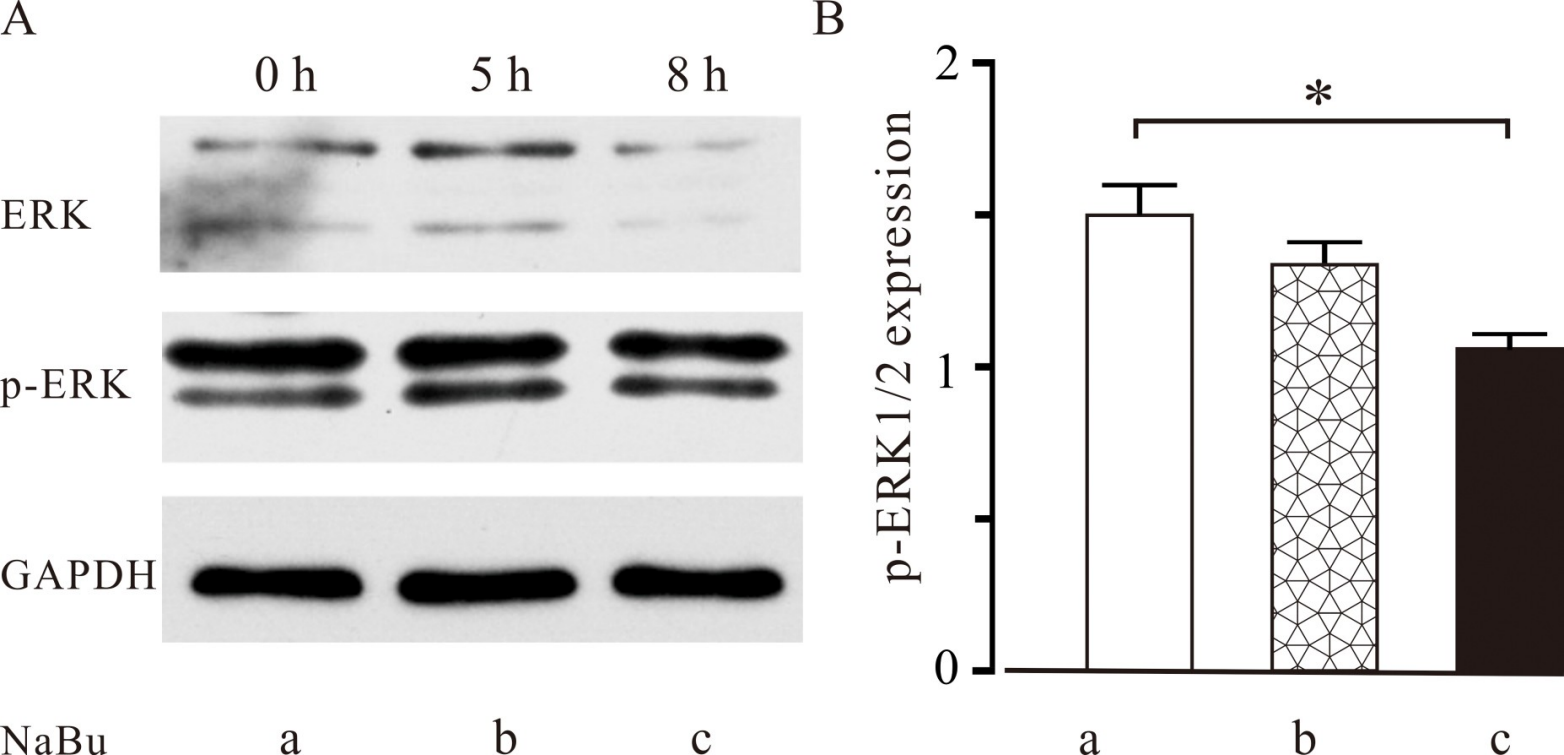
Effects of NaBu treatment on the expression of ERK and p-ERK in mouse oocytes at the MI stage. Mouse GV stage oocytes were collected and randomly assigned into three groups. GV stage oocytes were cultured in KSOM without NaBu for 8 h as a negative control (group (a)). In group (b), GV stage oocytes were cultured in KSOM for 3 h followed by culturing in KSOM supplemented with 2.0 mM NaBu for 5 h. In group (c), GV stage oocytes were cultured for 8 h. After 8 h of culture, the oocytes at the MI stage in these three groups were lysed to extract their total proteins. Then, western blotting was performed to detect to expression of p-ERK and ERK in groups a (left), b (central), and c (right). (B) Summary of ratios of p-ERK/ERK in (A). *: *P* < 0.05. These data suggest that NaBu treatment reduced the ratio of p-ERK/ERK in MI stage oocytes.

Previous studies have reported that p-ERK and ERK play an important role in the maturation of oocytes. Therefore, to elucidate the underlying mechanisms by which NaBu induces abnormalities in the spindle apparatus of oocytes, the expression levels of p-ERK and ERK were measured. As shown in **Fig 3**, the western blotting results revealed that two bands (extracellular signal-regulated kinase 1/2) for both-ERK and p-ERK were detected. The ratio of p-ERK/ERK in group b was not significantly different compared with that of the control group a. Meanwhile, the ratio of p-ERK/ERK in group c significantly decreased compared with that of control group a (*P* < 0.01). However, the ratios of p-ERK/ERK of these two experimental groups were not significantly different. These data indicate that long-term NaBu treatment may result in a decrease in the p-ERK/ERK ratio in mouse oocytes.

### Effects of NaBu on dynamic changes in H3K9ac in mouse oocytes during maturation

**Fig 4:**
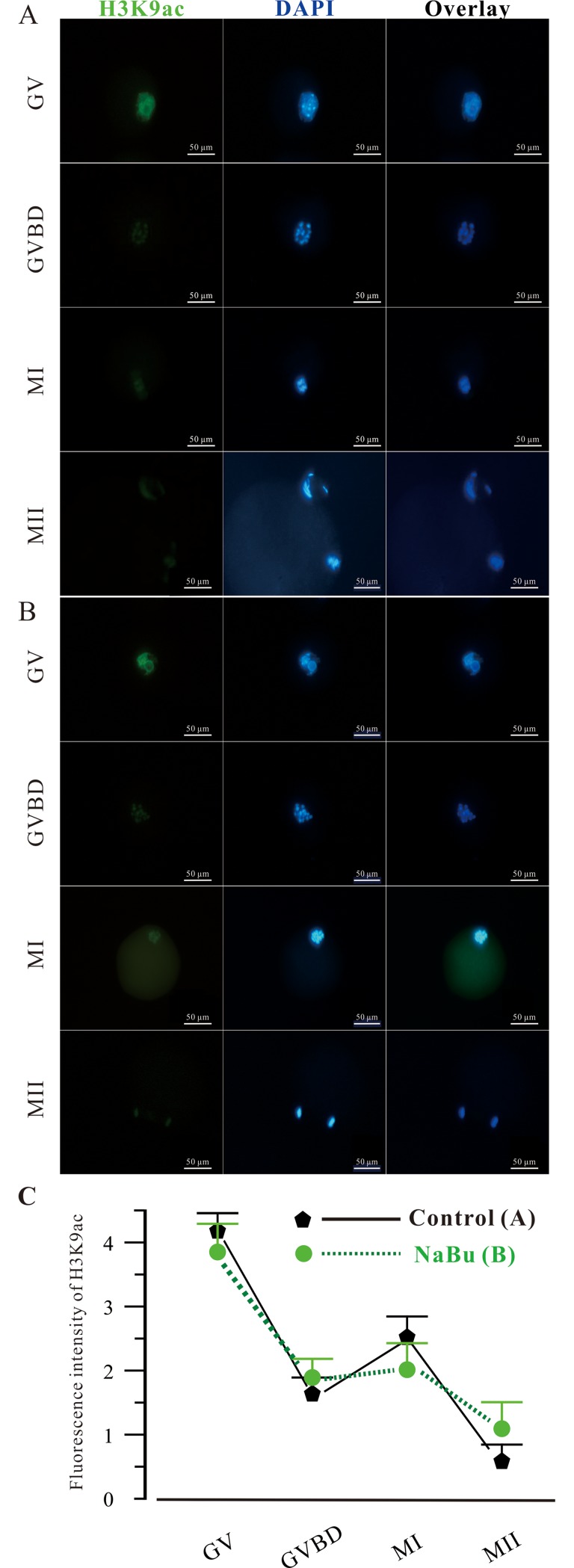
Effects of NaBu on dynamic changes in H3K9ac during the maturation of mouse oocytes. Mouse GV stage oocytes were obtained and cultured in KSOM without/with 2 mM NaBu in vitro. The cultured oocytes were collected at the GVBD, MI, and MII stages to analyze the expression level of H3K9ac using immunofluorescence techniques. The oocytes at different stages were immunostained using anti-H3K9ac antibody (green). The DNA was counterstained using DAPI (blue). Scale bar: 50 μM. (A) Control group, (B) NaBu group, and (C) summary of H3K9ac expression in (A) and (B).

The effects of NaBu dynamic changes in the acetylation levels of H3K9 protein in the maturation of oocytes were explored using immunofluorescence. As demonstrated in **Fig 4**, the acetylation levels of H3K9 fluctuated during the entire maturation process of oocytes *in vitro*. In GV stage oocytes, the acetylation level of H3K9 reached a high level (4.26 ± 0.92). In GVBD stage oocytes, the acetylation level of H3K9 significantly decreased (1.60 ± 0.92, *P* < 0.05). Unexpectedly, the acetylation level of H3K9 proteins in MI oocytes slightly increased compared with that in GVBD stage oocytes. Finally, in MII stage oocytes, the expression levels of H3K9ac decreased to a level that was significantly lower than that of GVBD stage oocytes. Among the four stages, NaBu treatment resulted in no significant differences in the expression levels of H3K9ac. These data suggest that the general tendency of the acetylation levels of oocytes during *in vitro* maturation is to decrease. In the GV stage, the expression level of H3K9ac was highest, while the level dropped to the lowest level at the MII stage. NaBu treatment did not affect the expression levels of H3K9ac during oocyte maturation *in vitro*.

### Dynamic changes in H3K9ac in mouse embryos derived from *in vivo*, parthenogenesis (Ps), IVF, and IVF+NaBu

**Fig 5:**
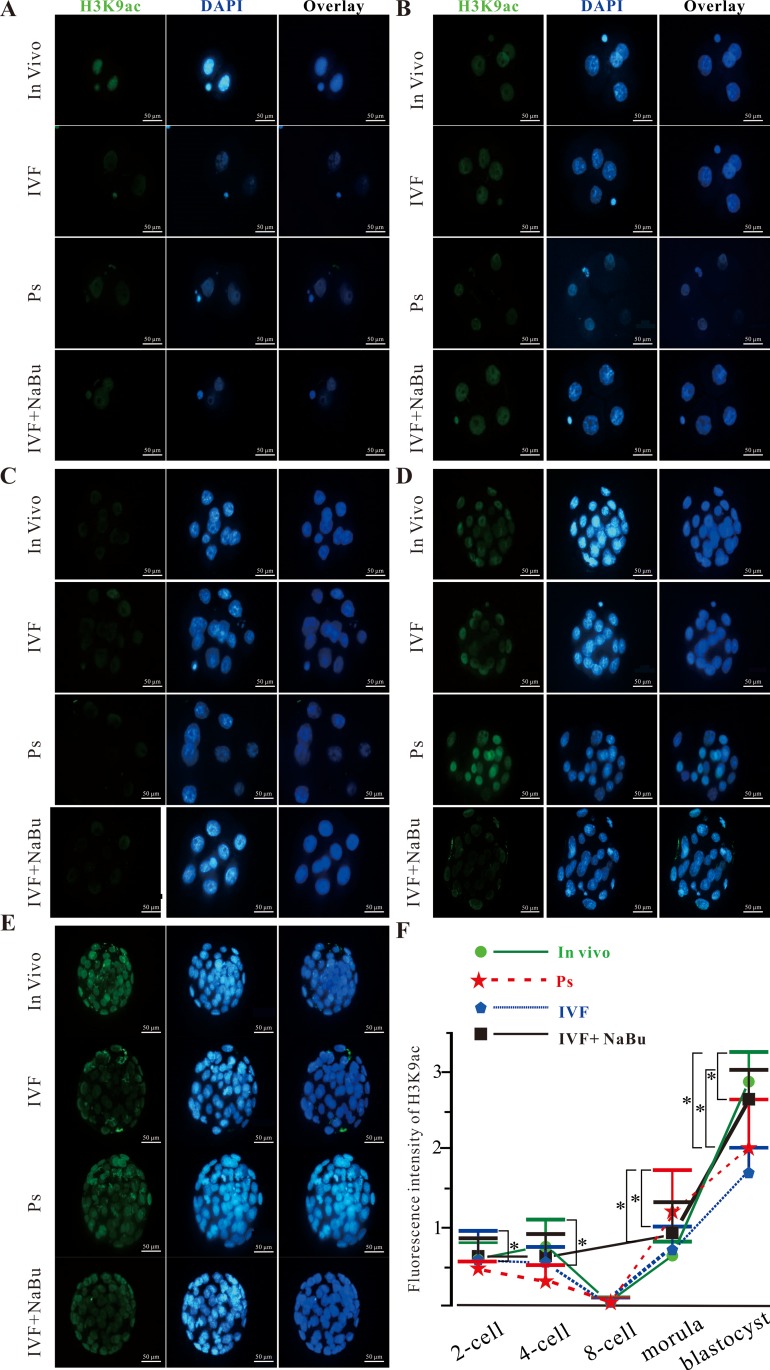
Acetylation status of H3K9 during the preimplantation development of embryos derived from *in vivo*, Ps, IVF, and IVF+NaBu. Embryos derived from *in vivo*, Ps, IVF, and IVF+NaBu were obtained to analyze the expression of H3K9ac using immunofluorescence technology. Immunofluorescent images of embryos at the 2-cell (A), 4-cell (B), 8-cell stage (C), morula (D), and blastocyst (E) stages that were immunostained using anti-H3K9ac antibody (green). The DNA was counterstained with DAPI (blue). (F) Summary data of H3K9ac fluorescence in (A-E). *: *P* < 0.05.

The dynamic changes in H3K9 acetylation levels in embryos derived from natural mating, IVF, and Ps were determined and calculated using immunofluorescence. As shown in **Fig 5**, in the 2-cell stage, the expression level of H3K9ac (0.76 ± 0.32) in the IVF group was higher than those of the *in vivo* (0.54 ± 0.25) group and Ps group (0.48 ± 0.14). However, there was no significant difference among these three groups (*P* > 0.05). In the 4-cell stage, the expression level of H3K9ac in the IVF and Ps groups decreased compared with those of their counterparts in the 2-cell stage. On the contrary, the expression level of H3K9ac in *in vivo* embryos increased. The acetylation level of H3K9 (0.76 ± 0.27) in the *in vivo* group was significantly higher than that in the Ps group (0.28 ± 0.20) (*P* < 0.05). In the 8-cell stage, the expression level of H3K9ac in these three groups all decreased to their lowest points. In the morula stage, the acetylation levels of H3K9 in these three groups all increased. Meanwhile, the expression level of H3K9ac in the Ps group was significantly higher than those in the *in vivo* and IVF groups (*P* < 0.05). In the blastocyst stage, the expression level of H3K9ac in the *in vivo* group was significantly higher than those in the IVF and Ps groups. Meanwhile, NaBu treatment resulted in a significant increase in the H3K9ac expression level compared with that in the IVF group (*P* < 0.05). In conclusion, the expression of acetylated H3K9 in *in vivo*, IVF, and Ps embryos decreased from the 2-cell stage to the lowest level at the 8-cell stage, followed by an increase in the morula and blastocyst stages. NaBu treatment can increase the level of acetylated H3K9 in blastocysts derived from IVF.

### Effects of NaBu on embryonic developmental potential *in vitro*

To further explore the effects of NaBu on mouse embryos, the effects of NaBu on the developmental competence of embryos were explored. The IVF embryos were randomly divided into two groups: one supplemented with 0 mM (control) NaBu and one supplemented with 2.0 mM (experimental) NaBu. As shown in **Table 3**, the rates of embryos in the experimental group at the 2-cell, 4-cell, and morula stages were higher than those of their counterparts in the control group (*P* > 0.05). Meanwhile, the average cell numbers of blastocysts in the NaBu-treated group significantly increased compared with those in the control group (*P* < 0.05). However, the blastocyst rate in the experimental group was significantly higher than that in the control group (*P* < 0.05). These data suggest that NaBu treatment may be beneficial in embryonic development *in vitro*.

**Table 3:** Effects of NaBu on the development of mouse embryos *in vitro*.

Groups	No. of zygotes	Treat time (h)	Embryo development rates (%)				Total cell number
			2-cell	4-cell	morula	blastocyst	
Control	131	0	74.6 ± 5.4	52.7 ± 2.8	29.7 ± 1.9	14.7 ± 2.1(19)^a^	72.3±7.2^a^
NaBu	136	24	80.1 ± 3.2	58.4 ± 4.1	34.4 ± 3.4	20.6 ± 2.6(32)^b^	80.2±6.3^b^

Different letters in the same column indicate significant differences (*P < 0*.*05*).

### Effects of NaBu on the mRNA expression of important developmental genes

**Fig 6:**
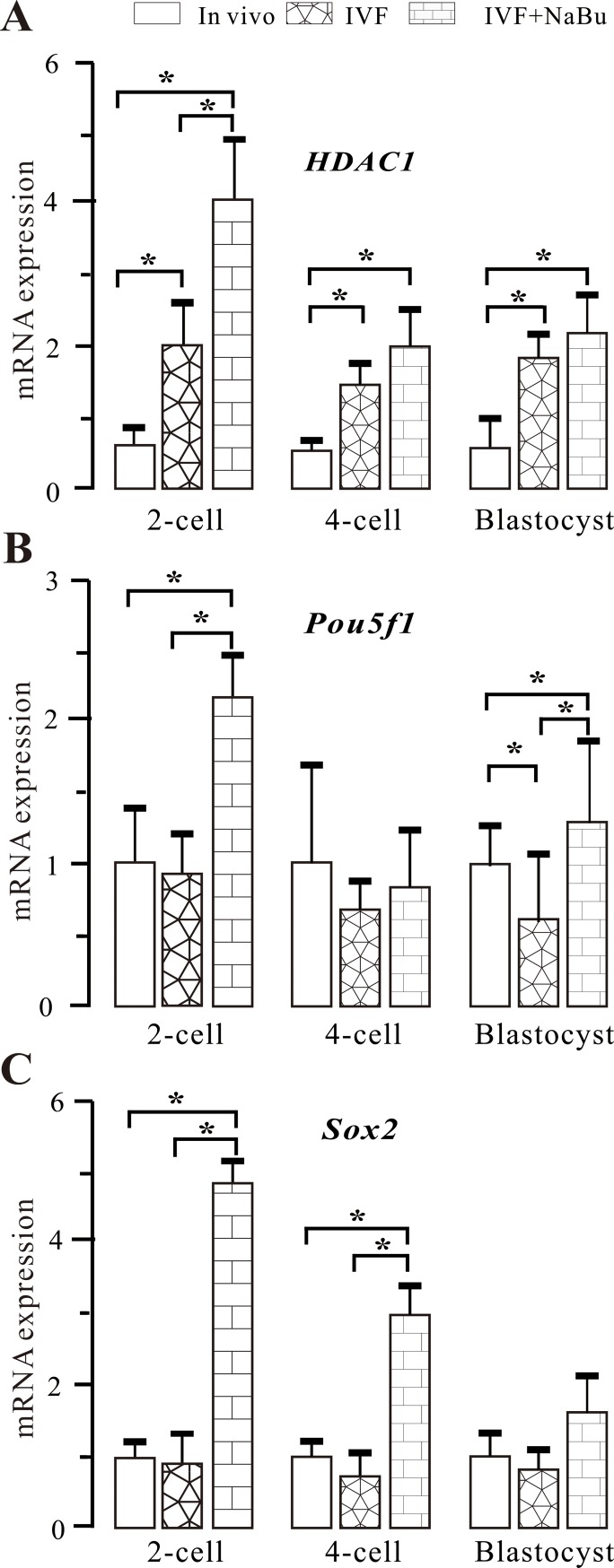
Expression profiles of *HDAC1*, *Pou5f1*, and *Sox2* in mouse embryos during preimplantation development. Mouse embryos derived from *in vivo* and IVF at the 2-cell, 4-cell, and blastocyst stages were collected and used for RT-PCR; these embryos were divided into three groups: the *in vivo*, IVF, and IVF+NaBu groups, respectively. (A) Comparative expression levels of HDAC1 mRNA. *: *P <* 0.05. (B) Comparative expression levels of Pou5f1 mRNA. *: *P <* 0.05. (C) Comparative expression levels of Sox2 mRNA. *: *P <* 0.05.

Both development-related pluripotent genes such as *Pou5f1* and *Sox*2 and the histone acetylation-related gene *HDAC1* play important roles in embryonic development. To elucidate the effects of NaBu on embryonic development, the mRNA expression levels of these three genes were measured using real-time PCR technology. IVF-derived embryos at the 2-cell, 4-cell, and blastocyst stages were collected and randomly placed into two groups: the IVF and IVF+NaBu groups. Embryos derived from *in vivo* were used as a control. As shown in **Fig 6**, for the *HDAC1* gene, the mRNA expression levels in both the IVF and IVF+NaBu groups were significantly higher than that in the control group (*P* < 0.05). Meanwhile, its expression in the NaBu-treated group was also significantly higher than that in the control group (*P* < 0.05). For the *Pou5f1* gene, NaBu treatment led to a significant increase in both the 2-cell and blastocyst stages compared with the values in the *in vivo* and IVF groups (*P* < 0.05). For the *Sox2* gene, NaBu treatment led to a significant increase in both the 2-cell and 4-cell stages compared with the values in the *in vivo* and IVF groups (*P* < 0.05). These data indicate that NaBu treatment has beneficial effects on the expression of development-related genes.

## Discussion

This study aimed to explore the effects of NaBu on the maturation of GV oocytes, preimplantation embryonic development, and the mRNA expression of pluripotent genes. Our data indicate that NaBu treatment of GV oocytes reduced the rates of GVBD and PBE in a dose-dependent manner. The level of ERK phosphorylation (p-ERK1/2) in NaBu-treated oocytes significantly decreased compared with that in the control group. Meanwhile, NaBu treatment significantly increased the rates of blastocyst formation and acetylated H3K9ac protein compared with their counterparts in the control group. Taken together, 2.0 mM NaBu was beneficial for early embryonic development in mice.

Previous studies have reported that the poor efficacy of SCNT and developmental defects in SCNT embryos are due to inadequate or abnormal reprogramming of somatic cell nuclei. Therefore, to facilitate nuclear reprogramming, considerable efforts have been made to improve SCNT efficacy via treating donor cells using epigenetic drugs such as HDACi TSA [15] alone or in combination with the DNA methyltransferase inhibitor 5-aza-2'-deoxycytidine [16]. However, some researchers reported that HDACi treatment did not alter or impair the development of embryos [22]. There is considerable evidence to suggest that the discrepancy regarding whether HDACi treatment improves SCNT efficacy is due to the type of HDACi and animal species. NaBu is a natural product of the microbial fermentation of dietary fiber in the colon and has been widely explored for improving the efficacy of epigenetic reprogramming and developmental competence of preimplantation embryos. The accumulated evidence indicates that NaBu improves SCNT efficacy via increasing the acetylation level of donor cells and/or SCNT embryos. However, little attention has been paid to the effects of NaBu on the maturation of oocytes.

Our data demonstrated that NaBu treatment reduced the rate of PBE of GV oocytes in a dose-dependent manner (**Table 2 and Fig 1**). These results suggest that NaBu treatment will have a harmful effect on the meiosis of oocytes. This is consistent with the previous study by Liu et al., in which they reported that NaBu can delay the meiosis of oocytes in both the GV and GVBD stages in an exposure-dependent manner in porcine oocytes [38]. The harmful effects of NaBu on meiosis is similar to those of TSA, another HDACi. Previous studies have reported that TSA can delay the onset of GVBD [39] and even result in the complete inhibition of GVBD in pigs [40]. However, a discrepancy exists regarding the harmful effects of TSA on oocyte meiosis. Kim et al. reported that TSA resulted in no meiotic blockage in mouse oocytes, which may be attributed to species differences [41]. In this study, the results of the immunofluorescence analysis of the spindle apparatus shown in **Fig 2** demonstrated that NaBu interrupted the separation of chromosomes in MI oocytes, suggesting that NaBu disrupted meiosis in mice. The potential harmful effects of NaBu on oocyte maturation were further supported by the western blotting results shown in **Fig 3** that indicated that GV oocytes exposed to NaBu for a longer time downregulated their expression of p-ERK, which is required to activate MAPKs. In turn, low MAPK activation will attenuate the maturation of oocytes via inhibiting the release of intracellular Ca^2+^ [42]. Therefore, in order to minimize the potential harmful effects of NaBu on embryonic development, a relative low concentration of NaBu (2.0 mM) was used in the following experiments.

The acetylation of histones is performed by histone acetylation transferases at the conserved lysine residues of the N-terminal tails of histones. There are at least two highly conserved lysine (K) residues in histone H3 (K9, K14) and four in histone H4 (K5, K8, K12, and K16) [43]. H3K9, one of these highly conserved lysines, was analyzed*to determine the effects of NaBu on its acetylation*. Accumulated lines of evidence suggest that individual acetylated histones, even particular acetylated residues of the same histone, may result in specific functional effects via changing the conformation of chromatin or regulating the interaction between proteins and histones. The immunofluorescence results shown in **Fig 4** and **Fig 5** demonstrated that the expression of H3K9ac during maturation and early embryonic development before the 8-cell stage showed a tendency to decrease, which will aid in erasing the epigenetic memory in sperm/oocytes and driving the transitional activation in the maternal-to-zygotic transition. Meanwhile, NaBu treatment did not affect the expression levels of H3K9ac during oocyte maturation *in vitro*. However, the expression levels of H3K9ac in the Ps group were significantly different compared with those in *in vivo* or IVF group at the 2-cell, 4-cell, morula, and blastocyst stages. This may be a possible explanation for the poor developmental competences of Ps embryos. Meanwhile, the expression levels of H3K9ac in all three groups reached their lowest levels at the 8-cell stage. Based on these results, to improve the developmental competence of mammalian embryos, the usage of an HDACi before the 8-cell stage may be a better choice in mice. Interestingly, NaBu treatment significantly increased the expression level of acetylated H3K9 at the blastocyst stage (**Fig 5**), which may be helpful in the early development of preimplantation embryos (**Table 3**). Further investigations are needed to determine the profile of H3K9ac dynamic changes in preimplantation embryos in other mammals.

The onset of transcription during the activation of the zygotic genome plays a crucial role in early embryogenesis. Previous studies have reported that incorrect and/or incomplete transcription is the main reason for low efficacy in SCNT embryos [44]. During transcription activation, a large number of genes are activated, and there is extensive reprogramming of embryonic gene expression, including of important developmental genes. Consequently, we measured the expression patterns of *HDAC1*, *Sox2*, and *Pou5f1*. *HDAC1*, a member of the histone deacetylases, is primarily responsible for the steady-state level of histone acetylation and participates in the regulation of development and gene expression during the development of mouse preimplantation embryos [45]. *Sox2* controls the transcriptional networks required for pluripotency in collaboration with *Pou5f1*, and *Nanog* and participates in the regulation of early embryonic development. In this study, NaBu treatment resulted in a significant increase in *HDAC1* mRNA in the 2-cell, 4-cell, and blastocyst stages compared with those in the untreated counterparts in the *in vivo* and IVF groups (**Fig 6**). The increase in *HDAC1* will enhance the acetylation level of histones, which in turn will promote the developmental competence of embryos. Meanwhile, the mRNA of the *Pou5f1* and *Sox2* genes in the NaBu-treated group was significantly upregulated, which is beneficial for the development of preimplantation embryos (**Table 3**). In general, NaBu treatment upregulated the mRNA expression of important developmental genes such as *HDAC1*, *Pou5f1*, and *Sox2* and enhanced the quality of blastocysts derived from IVF.

In summary, our results suggest that NaBu treatment of GV oocytes reduced the rates of GVBD/PBE and interrupted the separation of chromosomes during maturation. However, NaBu treatment improved the developmental competence of embryos *in vitro* and enhanced the mRNA expression of important developmental genes. Further investigations are still needed to determine the direct or indirect effects of NaBu on the regulation of important developmental genes and their subsequent impacts on full-term development.
