# Correction: Sodium butyrate interrupts the maturation of oocytes and enhances the development of preimplantation embryos

**DOI:** 10.1371/journal.pone.0221348

**Published:** 2019-08-14

**Authors:** Meng-Fei Yu, Ju-Long Wang, Jian-Ming Yi, Lin Ma

An additional affiliation is missing for the fourth author. Lin Ma is also affiliated with State Key Laboratory of Freshwater Ecology and Biotechnology, Institute of Hydrobiology, Chinese Academy of Sciences, Wuhan, China.
